# Repeat 24-hour recalls and locally developed food composition databases: a feasible method to estimate dietary adequacy in a multi-site preconception maternal nutrition RCT

**DOI:** 10.1080/16546628.2017.1311185

**Published:** 2017-04-11

**Authors:** Rebecca L. Lander, K. Michael Hambidge, Nancy F. Krebs, Jamie E. Westcott, Ana Garces, Lester Figueroa, Gabriela Tejeda, Adrien Lokangaka, Tshilenge S. Diba, Manjunath S. Somannavar, Ranjitha Honnayya, Sumera A. Ali, Umber S. Khan, Elizabeth M. McClure, Vanessa R. Thorsten, Kristen B. Stolka, For the Women First Preconception Nutrition Trial Group

**Affiliations:** ^a^ Department of Pediatrics, Section of Nutrition, University of Colorado School of Medicine, Aurora, CO, USA; ^b^ Department of Planning, INCAP (Institute of Nutrition of Central America and Panama), Guatemala City, Guatemala; ^c^ Department of Community Health, Kinshasa School of Public Health, Kinshasa, Democratic Republic of the Congo; ^d^ Women’s and Children’s Health Research Unit, KLE University’s Jawaharlal Nehru Medical College, Belagavi, India; ^e^ Department of Community Health Sciences, Aga Khan University, Karachi, Pakistan; ^f^ RTI International, Durham, NC, USA

**Keywords:** Dietary assessment, nutrition, pregnant women, low- and middle-income countries

## Abstract

**Background**: Our aim was to utilize a feasible quantitative methodology to estimate the dietary adequacy of >900 first-trimester pregnant women in poor rural areas of the Democratic Republic of the Congo, Guatemala, India and Pakistan. This paper outlines the dietary methods used.

**Methods**: Local nutritionists were trained at the sites by the lead study nutritionist and received ongoing mentoring throughout the study. Training topics focused on the standardized conduct of repeat multiple-pass 24-hr dietary recalls, including interview techniques, estimation of portion sizes, and construction of a unique site-specific food composition database (FCDB). Each FCDB was based on 13 food groups and included values for moisture, energy, 20 nutrients (i.e. macro- and micronutrients), and phytate (an anti-nutrient). Nutrient values for individual foods or beverages were taken from recently developed FAO-supported regional food composition tables or the USDA national nutrient database. Appropriate adjustments for differences in moisture and application of nutrient retention and yield factors after cooking were applied, as needed. Generic recipes for mixed dishes consumed by the study population were compiled at each site, followed by calculation of a median recipe per 100 g. Each recipe’s nutrient values were included in the FCDB. Final site FCDB checks were planned according to FAO/INFOODS guidelines.

**Discussion**: This dietary strategy provides the opportunity to assess estimated mean group usual energy and nutrient intakes and estimated prevalence of the population ‘at risk’ of inadequate intakes in first-trimester pregnant women living in four low- and middle-income countries. While challenges and limitations exist, this methodology demonstrates the practical application of a quantitative dietary strategy for a large international multi-site nutrition trial, providing within- and between-site comparisons. Moreover, it provides an excellent opportunity for local capacity building and each site FCDB can be easily modified for additional research activities conducted in other populations living in the same area.

## Introduction

The second United Nations Sustainable Development Goal for 2030 focuses on ending all forms of malnutrition, particularly by addressing the nutritional needs of women and children living in low-resource settings [1]. Certainly, in recent years great emphasis has been placed on targeted nutrition interventions during ‘The First Thousand Days’, the exceptionally vulnerable period from conception through two years of age when sub-optimal nutrition can result in long-term negative physical and developmental consequences, especially for those living in poverty [2,3].

Women of reproductive-age living in such settings are likely to be at risk of inadequate nutrient intakes, particularly of multiple micronutrients [4]. Yet quantitative dietary data generated from rural areas in low- and middle-income countries (LMIC) are sparse due to the intensive and complex work required to compile a robust food composition database (FCDB), as well as lack of validated regional database sources. In a strong effort to overcome the latter issue, steady work has been done over the past few years to improve the quality of several regional food composition tables (FCTs) as shown by the International Network of Food Data Systems (INFOODS) of the Food and Agricultural Organization (FAO) of the United Nations [5]. This work has been an invaluable contribution in providing a reliable framework for local FCDB development, particularly in regions of West/Central Africa and the Asian sub-continent. Yet only individual raw foods are typically included, adding to the complexity of estimating the correct nutrient composition of commonly consumed cooked foods.

Thus, as part of the Women First Preconception Nutrition Trial (WFPNT) [6], a large multi-site individually randomized controlled trial (RCT), our aim was to implement a robust dietary methodology in order to estimate the dietary adequacy and prevalence of the population ‘at risk’ of inadequate nutrient intakes in first-trimester pregnant women living in four LMIC. Furthermore, we plan to examine associations between dietary intakes and nutritional outcomes in this longitudinal study. To this end, we incorporated the use of repeat 24-hr recalls and the construction of a unique local FCDB at each of the four sites. This paper describes the dietary methods implemented here, including the advantages and challenges faced during the process.

## Methods

### Study design

Dietary assessment was planned for pregnant women living in rural areas of Democratic Republic of the Congo (DRC), Guatemala, India and Pakistan participating in the WFPNT from 2012–2017, as described earlier [6]. Briefly, the objective of this 3-armed RCT is to determine the benefits to the offspring of women in poor environments of commencing a daily comprehensive maternal nutrition supplement at least 3 months prior to pregnancy (Arm 1) versus commencing the same supplement at 12–14 week gestation (Arm 2) and to compare offspring outcomes with those not receiving supplement (Arm 3). From each of the four sites, 240 women (total 960) were randomized from Arms 1 and 2 to receive two 24-hr dietary recalls conducted 2–4 weeks apart once pregnancy was confirmed and prior to 12 week gestation. Of the ‘dietary’ women, half were randomized from Arm 1 and half from Arm 2, the latter group’s diet assessed prior to commencing the supplement.

A five-day training was provided at the beginning of the study by the lead study nutritionist (RL) for local nutritionist(s) at each site, except Pakistan, for whom the initial training was conducted in person in Colorado, USA. Based on recommendations from several key sources [7–10], training topics included: methodology of the multiple-pass 24-hr recall (i.e. revisiting and checking the dietary information during the interview); appropriate interview techniques; estimation of portion sizes; conversion of consumed amounts to gram weight equivalents; development of generic recipes for mixed dishes/beverages; and step-wise instructions to compile a site-specific FCDB. Process mentoring continued on a weekly basis by distance (via Skype and email) throughout the study.

### Assessment of food intakes

A food intake database was constructed based on two 24-hr recall interviews, standardized for all sites and pretested at each location. The dietary recalls were conducted by the site nutritionist on non-consecutive days within two weeks of each other in the participant’s home with the following information reported: participant ID; recall day; season; type of day (e.g. usual, feast, market, fasting); previous day’s health; food consumption time (e.g. breakfast, lunch, dinner or snack); location of food consumed (e.g. in the home, outside); food/dish name; food code; and amount consumed (grams). Continuous monitoring by the lead study nutritionist of all data entry provided checks to minimize reporting errors.

Locally developed picture charts served as an aid to prompt remembrance of foods consumed and food probe questions encouraged detailed descriptions of reported foods/beverages. For example, poultry probe questions included the kind of bird, exact part or piece, meat plus skin or meat only, method of cooking, bones (waste factor), etc. To estimate portion size, the participant provided the same amount of food or drink consumed the prior day using local spoons/containers and then weighed in grams. However, since left-over food was usually unavailable in these poorly-resourced households, photographs displaying a graduated range of portion sizes were used frequently to obtain the best estimation of portion amount, as well as reported number of spoon- or hand-fuls from the ‘family pot’. The site nutritionists were also very familiar with the local market value of commonly purchased items, e.g. 50 Congolese francs purchased approximately 30 grams of roasted peanuts.

**Figure 1.**
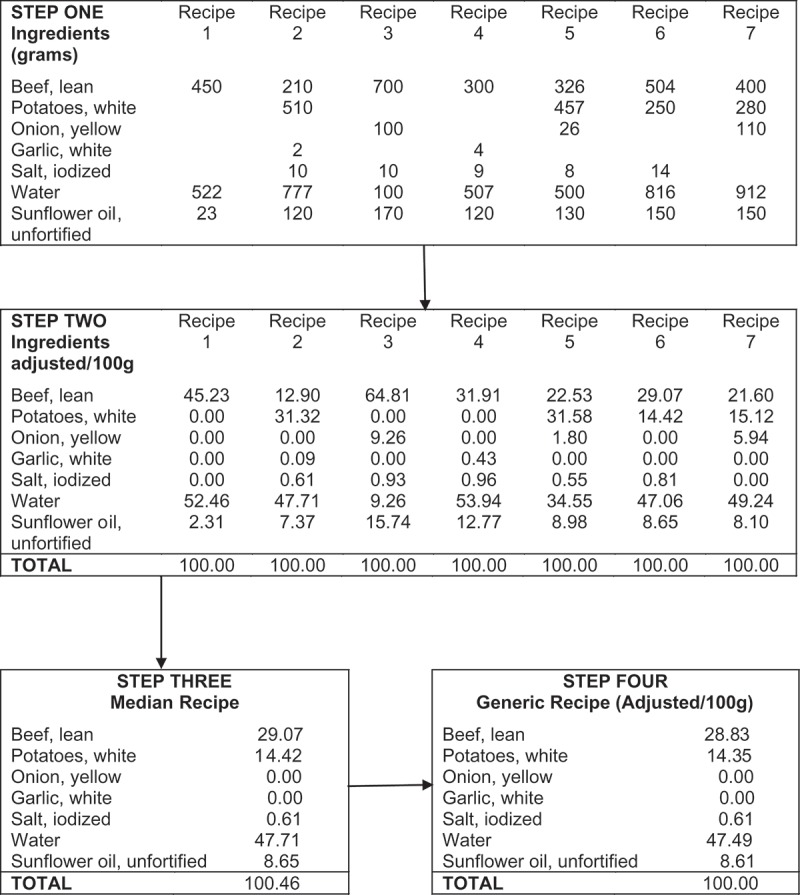
Construction of a generic recipe ‘beef stew with potatoes’.

Mixed dishes and beverages containing more than one ingredient were typically consumed by these populations. Thus, the development of local ‘generic’ representative recipes were used to calculate their nutrient content. For each mixed dish/beverage consumed by the study population, 5–10 local recipes were collected (amounts in grams) from several different women participants by the site nutritionist, with particular care to ensure water and oil added during cooking were also included in the recipe. In some situations (e.g. Pakistan), the local nutritionist watched the recipe preparations, measuring exact quantities (grams) of ingredients used in the dish. This process was followed by calculation of a median recipe per 100g (see Figure 1). All recipes were checked for feasibility and accuracy of calculations by the lead study nutritionist.

### Site-specific food composition database compilation

A unique FCDB was constructed at each site based on the food intake data collected from the dietary recalls. Food names were included in both the local language and in English, with careful description of the exact food. Food components included moisture content, energy, macronutrients (protein, total fat and fatty acids [saturated, monounsaturated, polyunsaturated], carbohydrate); dietary fiber; minerals (calcium, iron, zinc); vitamins including thiamine (B1), riboflavin (B2), pyridoxal phosphate (B6), folate (B9), cobalamin (B12), choline, betaine, ascorbic acid (C), and vitamin A (retinol activity equivalent, RAE); and an anti-nutrient (phytate). Appropriate folate values (either food folate or dietary folate equivalent, DFE) were chosen based on the country’s national fortification policies. All choline and betaine values were sourced from the current United States Department of Agriculture (USDA) National Nutrient Database for Standard Reference, Release 28 [11], or the latest USDA Database for the Choline Content of Common Foods [12].

The 13 designated food groups included: starchy tubers and staples; legumes and nuts; milk and dairy; organ meats; eggs; flesh foods (meat or poultry); fish and miscellaneous small animal protein (e.g. insects); vitamin A-rich vegetables and fruit (≥ 60 RAE/100g); other vegetables and fruit; fats and oils; sweets and sugars; beverages; and miscellaneous, e.g. fast foods. Generic recipes were assigned to the appropriate food group, depending on the primary ingredient or content of foods significantly affecting the overall nutrient content of the recipe, e.g. presence of animal-source foods.

FCDB raw and cooked nutrient values for DRC, India and Pakistan were primarily derived from recently developed FAO-supported regional FCTs, i.e. the West African FCT [13] was used for the DRC site FCDB whereas the Indian and Pakistan sites utilized the Bangladesh FCT [14]. For the Guatemala FCDB, values were primarily taken from the USDA database [11], as well as from the database compiled by the Institute of Nutrition of Central America and Panama (INCAP) [15], with care that food choices from the USDA database were consistent with the Guatemalan national food fortification policies. Finally, every effort was made to ensure the imputed values reflected the same analytical methodology across all databases.

Missing values from the West African or Bangladesh FCTs were usually borrowed from the USDA database with adjustments to account for differences in moisture content, unless a more precise food match was found in the World Food Dietary Assessment System (WFDAS) [16]. The latter also provided all phytate values; however, moisture adjustments using the WFDAS were not possible. Additionally, a food match code was provided for all FCDB values as described by FAO/INFOODS [17], indicating whether the imputed values were an exact or similar match to the required food item.

**Figure 2.**
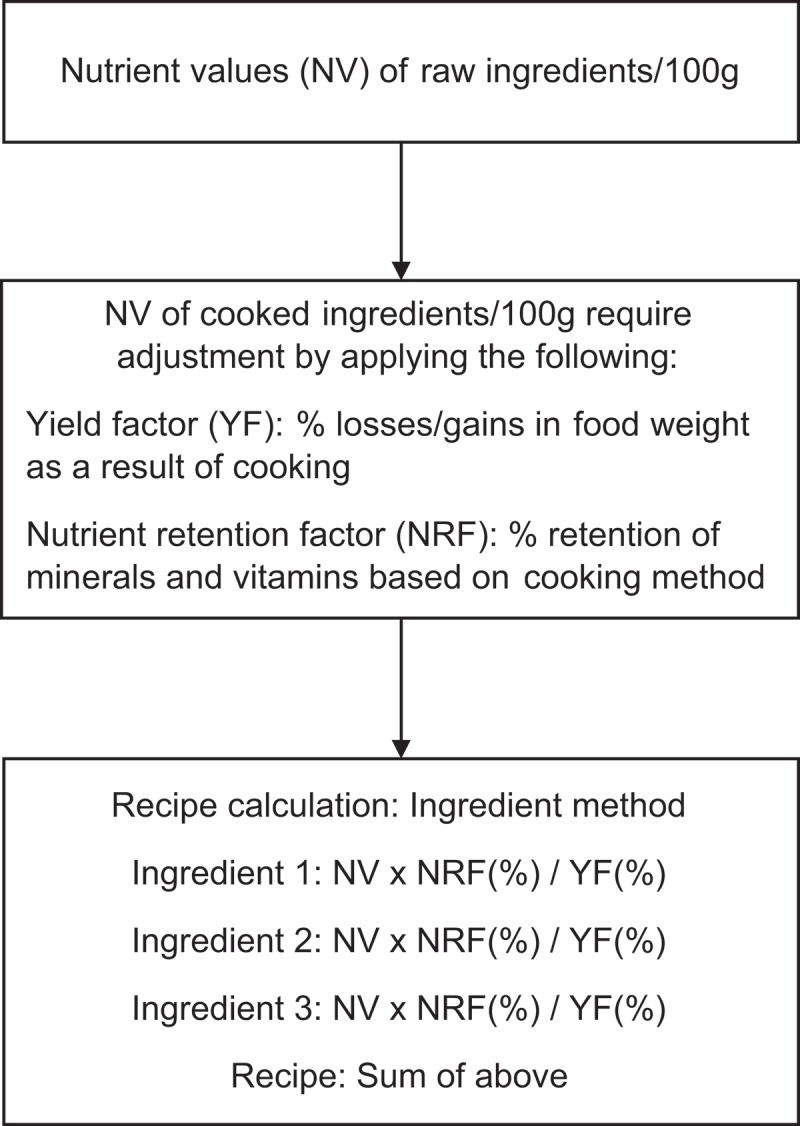
Method for generic recipe calculation at the ingredient level. (Charrondiere et al. 2011 [8])

For some foods, only raw values were available from the reference databases. Thus, when cooked values were required for individual foods and/or recipes, raw to cooked adjustments for nutrient retention and yield after cooking were made using appropriate retention [18] and yield factors [19,20]. These factors were applied at the ingredient level for the recipe nutrient calculations [8,9] (see Figure 2). In this method, the contribution of each ingredient to the overall recipe was calculated by multiplying the energy or nutrient value per 100g of each ingredient in its consumed form (i.e. raw or cooked) by its percentage contribution to the 100g generic recipe (i.e. ingredient contribution/100g), with the contribution from all ingredients added together. Then the ‘cumulative’ energy and nutrient values for each recipe were added into the appropriate database row.

Finally, a unique food code was assigned for every individual item and generic recipe row in each site FCDB. Upon completion, checks were planned according to FAO/INFOODS guidelines [21], ensuring no required foods or values were omitted.

### Statistical analyses

At the end of the WFPNT, this dietary method of conducting two 24-hr recalls in ≥100 women per arm will allow for analyses by site (overall and by arm) including: estimation of group usual energy and nutrient intake distributions, and determination of the percentage of the population ‘at risk’ of inadequate nutrient intakes [22] compared to international guidelines [23,24]. Within- and between-site comparisons will be made using a Student’s t test and analysis of variance (ANOVA), respectively, as well as multi-variate regression models examining associations between nutrient intake and relevant factors, e.g. maternal education, seasonal variation, etc. Analyses will utilize STATA statistical software package 13 (Stata corporation, College Station, TX, USA) and Intake Monitoring, Assessment and Planning Program (IMAPP) Version 1.0 (Iowa State University, 2010), with support from RTI International.

## Preliminary results

To date, two 24-hr recalls have been conducted in >200 women per site (~925 total), equally distributed between Arms 1 and 2. Further, >100 local generic recipes for mixed food/beverages have been developed per site. All site FCDBs are currently still in progress but thus far, each unique site FCDB contains between 260 to 350 foods, beverages, and recipes in total.

## Discussion

Our study has demonstrated that with adequate initial training and ongoing supervision, local nutritionists in four LMIC were able to conduct two standardized 24-hr recalls per dietary participant (>200 participants/site), as well as properly develop a site-specific FCDB. This method will allow for quantitative assessment of the diet in first-trimester pregnant women in these settings and their estimated risk of inadequate intakes. Furthermore, each unique FCDB can be readily modified for other population groups, e.g. children, adolescents, etc., in the future. Such a strategy is highly advantageous for large international RCTs, particularly in areas with ongoing research studies. This preconception trial, for example, is part of the Global Network (GN) for Women’s and Children’s Health Research, which has supported research activities and capacity building in these specific regions for more than a decade [25] and thus, the site FCDBs are likely to be useful for other studies in the future.

In recent years, the inclusion of dietary assessment as part of large nutrition research trials has gained greater priority, as demonstrated in the Etiology, Risk Factors and Interactions of Enteric Infections and Malnutrition and the Consequences for Child Health and Development (MAL-ED) study [26]. Typically, qualitative or semi-quantitative dietary methods (e.g. dietary history, food frequency questionnaire) have been used for the sake of cost and minimal participant burden. Yet proper validation of these instruments can prove challenging, especially in remote regions. Moreover, both of these methods are limited by the difficulty in estimating their inherent source of errors [27], and the within- and between-subject variability cannot be calculated [28]. In contrast, repeated weighed food records are the most precise method for estimating usual individual nutrient intakes, but it has a high respondent burden and is usually too expensive and cumbersome to be used in LMIC. However, the multiple pass 24-hr recall method has been used extensively in such settings with the advantage of relatively low participant burden [7]. Importantly, it can provide a reasonable reproducible estimate of the mean usual intakes of a group when conducted as done in this study (i.e. interviews on all days of the week with non-consecutive participant interviews) [28]. Furthermore, the inclusion of a repeat 24-hr recall for all participants allows the percentage of those ‘at risk’ of inadequate nutrient intakes to be calculated [22].

Such analyses are possible through the use of a robust local FCDB containing nutrient values for commonly consumed cooked mixed dishes and beverages. The inclusion of such complex foods, in addition to individual ingredients, allows for a more accurate estimate of nutrient intakes in these populations, especially when FCDB nutrient values are sourced from a high quality FCT with similar foods. Notably, the reference regional FCTs used here have all been published within the last five years, except for the INCAP and WFDAS databases. While compilation of a site-specific FCDB is typically considered beyond the scope of most research studies, we have demonstrated that local nutritionists can gain the necessary understanding and skills to complete this detailed task with comprehensive training and mentoring. We acknowledge a distinct advantage of this trial has been the continuous oversight provided by a well-trained lead nutritionist (RL), with ongoing coaching of local nutritionists and monitoring of all databases throughout the study.

However, challenges and limitations exist, especially in determining the correct nutrient values for local wild and cultivated edible foods consumed in remote geographical areas. In some instances, different wild species may have the same local name and thus some ambiguity remains as to the appropriate nutrient content of these foods. Organizations such as FAO/INFOODS and Bioversity International (www.bioversityinternational.org) are committed to identifying and publishing nutrient values for biodiverse foods [29]. Likewise, more high quality regional FCTs are urgently required to ensure the nutrient values chosen for local foods are accurate. Again, FAO/INFOODS and other groups (e.g. International Dietary Data Expansion Project, INDDEX) are making a concerted effort to expand the work of developing regional FCTs of high standard. Lastly, accurate determination of portion size was often very challenging in our study areas, particularly with food consumed from the family pot. Yet through the use of a variety of memory aids (e.g. graduated portion size photographs) and excellent familiarity with local household and market measures, we strove to gain the most reliable information possible on amounts consumed by participants.

Additionally, while every effort was made to ensure the nutrient values sourced between the site FCDBs reflected the same analytical methodology, a few discrepancies exist between databases. For example, total fat was determined by the mixed solvent extraction method in the USDA database [11] and most foods found in the West African FCT [13], whereas the Soxhlet method was used for fat values in the Bangladesh FCT [14] and for a few West African FCT values [13]. Finally, more than two 24-hr recalls might improve assessment of group intakes of certain micronutrients, which may have greater variability in the day-to-day diet. A recent Mexican study, with data from 31 cities, reported three 24-hr recalls, rather than one recall, improved the estimation of micronutrient intakes in these urban populations [30]. No comparison was made, however, between two versus three dietary recalls and as our study was conducted in poor rural communities, we anticipate the within-subject variation will be lower than the between-subject variation due to the limited number of the foods consumed in these very resource-poor environments [31].

In conclusion, we developed a feasible and practical quantitative dietary assessment method that affords the potential to gain much needed insight into the nutritional dietary adequacy of first-trimester pregnant women in these settings, allowing for within- and between-site comparisons. Incorporation of such dietary methodologies into large multi-site RCTs could be highly useful, especially in areas with ongoing research, as it provides an excellent opportunity for local capacity building, and the site FCDB can be easily modified for the specific population group of interest. Moreover, the application of this knowledge could help to refine targeted interventions, as well as provide a strong research base in order to more fully understand the dietary contribution to risk for other health outcomes and diseases in low-resource environments.

## References

[CIT0001] United Nations (2015). 2030 sustainable development goal 2: end hunger, achieve food security and improved nutrition and promote sustainable agriculture. https://sustainabledevelopment.un.org/sdg2.

[CIT0002] Piwoz E, Sundberg S, Rooke J. (2012). Promoting healthy growth: what are the priorities for research and action?. Adv Nutr.

[CIT0003] Victora CG, De Onis M, Hallal PC (2010). Worldwide timing of growth faltering: revisiting implications for interventions. Pediatrics.

[CIT0004] Torheim LE, Ferguson EL, Penrose K (2010). Women in resource-poor settings are at risk of inadequate intakes of multiple micronutrients. J Nutr.

[CIT0005] (2016). Food and Agricultural Organization of the United Nations/International Network of Food Data Systems (FAO/INFOODS). http://www.fao.org/infoods/infoods.

[CIT0006] Hambidge KM, Krebs NF, Westcott JE (2014). Preconception maternal nutrition: a multi-site randomized controlled trial. BMC Pregnancy Childbirth.

[CIT0007] Gibson RS, Ferguson EL (2008). An interactive 24-hour recall for assessing the iron and zinc intakes in developing countries. HarvestPlusTechnical Monograph 8.

[CIT0008] Charrondiere R, Burlingame B, Berman S (2011). Food composition study guide, volumes 1 and 2.

[CIT0009] Food and Agricultural Organization of the United Nations/International Network of Food Data Systems (FAO/INFOODS). E-learning course on food composition data (2013). http://www.fao.org/elearning/#/elc/en/course/FCD.

[CIT0010] Greenfield H, Southgate DAT (2003). Food composition data.

[CIT0011] US Department of Agriculture (USDA) (2015). USDA national nutrient database for standard reference, release 28. https://ndb.nal.usda.gov/ndb/.

[CIT0012] Patterson KY, Bhagwat SA, Williams JR (2008). USDA database for the choline content of common foods, release 2.

[CIT0013] Stadlmayr B, Charrondiere UR, Enujiugha V (2012). West African food composition table. Rome: food and Agricultural Organization. http://www.fao.org/infoods/infoods/tables-and-databases/faoinfoods-databases/en/.

[CIT0014] Shaheen N, Rahim AT, Mohiduzzaman M (2013). Food composition table for Bangladesh.

[CIT0015] Institute of Nutrition of Central America and Panama (INCAP) (2007). Table of food composition of Central America.

[CIT0016] Bunch S, Murphy SP (1997). User’s guide to the operation of the WorldFood dietary assessment system, version 2.0.

[CIT0017] Food and Agriculture Organization/ International Network of Food Data Systems (FAO/INFOODS) (2012). FAO/INFOODS guidelines for food matching, version 1.2.

[CIT0018] US Department of Agriculture (USDA) (2007). USDA table of nutrient retention factors, release 6.

[CIT0019] Bognár A (2002). Tables on weight yield of food and retention factors of food constituents for the calculation of nutrient composition of cooked food (dishes).

[CIT0020] Rahim AT (2013). Food and foodways of Bangladesh: a note on recipe composition and eating principle.

[CIT0021] Food and Agriculture Organization/ International Network of Food Data Systems (FAO/INFOODS): FAO/INFOODS guidelines for checking food composition data prior to the publication of a user / table database, version 1.0. Rome: Food and Agriculture Organization (2012). http://www.fao.org/infoods/infoods/standards-guidelines/en/.

[CIT0022] Institute of Medicine (2000). Dietary reference intakes: applications in dietary assessment.

[CIT0023] Food and Agricultural Organization of the United Nations/World Health Organization /United Nations University (FAO/WHO/UNU) (2001). Human energy requirements: report of a joint FAO/WHO/UNU expert consultation.

[CIT0024] World Health Organization/Food and Agricultural Organization of the United Nations (WHO/FAO) (2004). Vitamin and mineral requirements in human nutrition.

[CIT0025] Koso-Thomas M, McClure EM (2015). The Global Network for women’s and children’s health research: a model of capacity-building research. Semin Fetal Neonatal Med.

[CIT0026] Caulfield LE, Bose A, Chandyo RK (2014). Infant feeding practices, dietary adequacy, and micronutrient status measures in the MAL-ED study. Clin Infect Dis.

[CIT0027] Beaton GH (1994). Approaches to analysis of dietary data: relationship between planned analyses and choice of methodology. Am J Clin Nutr.

[CIT0028] Gibson RS (2005). Principles of nutritional assessment.

[CIT0029] Food and Agriculture Organization/International Network of Food Data Systems (FAO/INFOODS) (2013). FAO/INFOODS food composition database for biodiversity, version 2.1 – BioFoodComp2.1.

[CIT0030] Shamah-Levy T, Rodríguez-Ramírez S, Gaona-Pineda EB (2016). Three 24-hour recalls in comparison with one improve the estimates of energy and nutrient intakes in an urban Mexican population. J Nutr.

[CIT0031] Persson V, Winkvist A, Ninuk T (2001). Variability in nutrient intakes among pregnant women in Indonesia: implications for the design of epidemiological studies using the 24-h recall method. J Nutr.

